# Dietary Strategies to Reduce Triglycerides in Women of Reproductive Age: A Simulation Modelling Study

**DOI:** 10.3390/nu15245137

**Published:** 2023-12-18

**Authors:** Nahal Habibi, Shalem Leemaqz, Jimmy Chun Yu Louie, Thomas P. Wycherley, Jessica A. Grieger

**Affiliations:** 1 Robinson Research Institute, The University of Adelaide, Adelaide 5005, Australia; nahal.habibi@adelaide.edu.au (N.H.);; 2 Adelaide Medical School, The University of Adelaide, Adelaide 5005, Australia; 3 Department of Nursing and Allied Health, School of Health Sciences, Swinburne University of Technology, Melbourne 3122, Australia; jimmylouie@swin.edu.au; 4 Alliance for Research in Exercise, Nutrition and Activity, Allied Health and Human Performance, University of South Australia, Adelaide 5000, Australia; tom.wycherley@unisa.edu.au

**Keywords:** Australia, dietary modelling, foods, triglycerides, health survey, linear programming

## Abstract

Many women of reproductive age have poor diet quality and are at higher risk of chronic diseases such as diabetes. Triglycerides are a critical risk factor for chronic diseases, and although they can be influenced by diet, there are minimal dietary intervention studies identifying key foods/food groups that reduce triglycerides. We performed data simulation modelling to estimate the potential reductions in fasting triglycerides that could be achieved by different dietary strategies in reproductive age women. The model was created using data from the 2011–2013 Australian Health Survey and incorporated various factors such as demographics, nutrient intake, and plasma biomarkers. Multiple linear regression analysis was conducted to estimate triglyceride levels, considering nutrient intake and pre-determined covariates. Dietary scenarios were developed, reducing the consumption of processed/ultra-processed foods, while increasing the intake of minimally processed foods like fruits, vegetables, fish, and nuts. A total of 606 women were included. Reducing processed foods by 50% plus increasing intakes of fruits (75–225 g/day), vegetables (75–225 g/day), or nuts (10–40 g/day) decreased triglycerides by up to 4.3%. Additionally, incorporating 80 g/day of omega 3 fish (>800 mg long-chain omega 3/100 g) decreased triglycerides by 8.2%. The clinical relevance of lowering triglycerides for cardiometabolic disease management should be tested in dietary intervention studies in women.

## 1. Introduction

Reproductive age typically refers to all women aged 15–49 years, and includes the preconception, pregnancy, and postpartum periods [1]. The diet quality of many women in this age group is typically inadequate, attributed to a low intake of fruits and vegetables and higher intakes of discretionary foods containing added sugar, sodium, and saturated fat [2,3]. Furthermore, women of reproductive age have demonstrated the greatest rise in the prevalence of obesity [4], which is associated with increased risk of chronic diseases such as type 2 diabetes and cardiovascular disease [5,6].

Dyslipidaemia also has health consequences in reproductive age women; for example, women with polycystic ovary syndrome have higher levels of triglycerides, LDL-C, and non-HDL-C than controls, irrespective of BMI [7], and women with cardiometabolic risk factors are more likely to be infertile [8]. In pregnant women, we [9,10], and others [11,12,13], have demonstrated that raised triglycerides are a key risk factor for gestational diabetes, and in non-pregnant women of reproductive age, triglycerides are a risk factor for type 2 diabetes [14,15,16]. Furthermore, infertility [17], polycystic ovary syndrome [18], and gestational diabetes [19] can contribute to the development of type 2 diabetes and/or CVD in later life. Although nutritional intake influences lipid profile, there are no clearly defined dietary patterns for reproductive aged women that are recommended to be effective for such conditions [20,21,22].

Importantly, excess consumption of industrially processed foods is a driving factor regarding the increase in prevalence of diet-related chronic diseases [23]. Ultra-processed foods have been shown to contribute up to 60% of total daily energy intake in adults [24,25], and they are often made from cheap ingredients and additives [26]. We previously showed that in a sample of reproductive aged women, reducing ultra-processed foods (which made up 41% of total daily energy intake) by 50%, lowered total daily energy intake by 689 kJ (404 kcal) and reduced estimated intakes of saturated fat, added sugar, and sodium by 20–40% [27]. Little is known, however, about the association between lipids, specifically triglycerides, and ultra-processed foods in women of reproductive age.

Dietary modelling provides an opportunity to explore the link between changes in dietary factors, particularly lowering ultra-processed foods, on nutritional intake and/or health parameters. The current modelling aims to investigate the theoretical potential of different combinations of foods to lower triglycerides in reproductive aged women who participated in the Australian Health Survey. Raised triglycerides are a strong risk factor for several reproductive conditions, but there are minimal dietary intervention studies identifying key foods/food groups to reduce them [28]. Understanding how dietary intake might influence triglycerides is clinically relevant so that interventions can be devised for high-risk subgroups of women.

## 2. Materials and Methods

### 2.1. Study Design and Data Sources

This was a simulation modelling study based on a static, microsimulation, discrete time deterministic model. Data from the National Nutrition and Physical Activity Survey (NNPAS) 2011–2012, which is a part of the 2011–2013 Australian Health Survey [29], were used. This is the largest and most recent Australian dataset combining nutrition and biomedical information. The Census and Statistics Act, 1905 provided the Australian Bureau of Statistics with the authority to conduct the NNPAS. The NNPAS applied a complex, stratified, multistage probability cluster sampling design with selection of strata, households, and people within households to survey a randomly selected national sample of the Australian population (*n* = 12,153). Written informed consent was collected from all respondents. The current study used data from reproductive aged women (19–50 years) who provided a fasting blood sample (for assessment of plasma glucose and triglycerides, and high-density lipoprotein), and who had completed the first-24-hour food recall (*n* = 606) (Figure S1). Blood samples were collected at collection clinics or via a home visit using standard operating procedures for phlebotomy collection. Table S1 describes the characteristics of included and excluded participants, whereby the women included (606/2146) were approximately 1.5 years older in age, had a similar BMI, and slightly lower triglyceride (~0.1 mmol/L). This research was supported by the NHMRC (APP2000905), but the NHMRC had no input into this manuscript. The study follows the STROBE reporting checklist (Online Supporting Material).

The Food Standards Australia and New Zealand nutrient database (AUSNUT 2011–2013) [30] was used to estimate the energy and nutrient values per 100 g food from the 2011–2012 NNPAS, with further inclusion of added and free sugars [30] and glycaemic index and glycaemic load [31]. Based on the NOVA food classification, which is the most specific and comprehensive system for classification of foods based on food processing level [32], each food was allocated to one of the following groups: unprocessed/minimally processed foods (2182 foods), processed culinary ingredients (109 foods), processed foods (1369 foods), or ultra-processed foods (2081 foods). The population average nutrient intakes per person per day were estimated and used for scenario simulations. Scenarios were conducted at the population level and were modelled on grams of intake with the replacement (increase or decrease) of equivalent grams from other foods/food groups.

### 2.2. Dietary Scenarios

Table 1 details the rationale for the dietary scenarios and models.

Scenario 1: reducing NOVA processed and ultra-processed foods (PFs).

The first scenario simulated a 50% reduction in the gram weight of the NOVA processed foods and ultra-processed foods (collectively termed processed foods, PFs), i.e., a reduction in the average intake of each processed food item in the sample population. This strategy was designed to determine the effect of reducing foods that are high in energy and discretionary nutrients such as saturated fatty acids (SFAs), sugar, and sodium on population triglyceride level. This is the base model and is included in all subsequent scenarios except scenario six (oils).

Scenario 2: replacing PFs with NOVA unprocessed/minimally processed foods.

To examine the estimated impact of a 50% reduction in PFs (as described in Scenario 1, the base model) while concomitantly increasing intake of a broad range of unprocessed/minimally processed foods, we modelled a 25% (model 1), 50% (model 2), and 75% (model 3) increase in the NOVA unprocessed/minimally processed foods.

Scenario 3: replacing PFs with omega-3-containing foods.

In conjunction with a 50% reduction in PFs (Scenario 1), Scenario 3 estimated the impact of an incremental increase in high-omega-3 fish and seafood, from 40 g/d up to 200 g/d (Scenario 3a, model 1–5), or nuts, from 10 g/d up to 40 g/d (Scenario 3b; model 1–4). High-omega-3 fish and seafood were considered those containing ≥800 mg long-chain omega 3 fatty acids per 100 g, such as salmon, rainbow trout, and sardines.

Scenario 4: replacing PFs with fruits and vegetables.

Along with a 50% reduction in PFs, Scenario 4 tested an incremental increase in the gram intake of vegetables by 75 g/d (equivalent to 1 serving of vegetables) up to 300 g/d (model 1), fruits by 75 g/d (equivalent to half a serving of fruits) up to 300 g/d (model 2), and their combination (model 3).

Scenario 5: replacing PFs with different combinations of food choices in Scenarios 2–4.

Scenario 5 simulated the effect of different combinations of potentially feasible increases in nut (10–40 g/d), vegetable (75–225 g/d), and fruits (75–225 g/d) intake, and then the addition of high-omega-3 fish (40–120 g/d).

Scenario 6: replacing oils higher in saturated fatty acids with oils higher in poly- or monounsaturated fatty acids.

Scenario 6 estimated the impact of gradually replacing oils with high SFA content, such as butter, dairy blend, and margarine spread, with healthier alternatives including flaxseed, olive, canola, and sesame oils. This scenario did not include the base model, Scenario 1.

### 2.3. Modelling and Analysis

Multiple linear regression was performed on the data from participants with available blood samples to estimate triglyceride levels (*n* = 606). The regression model included the following covariates: age, BMI, ethnicity, family history of diabetes, smoking status, dietary intakes (energy, macronutrients, micronutrients, vitamins and minerals, fatty acids, alcohol, glycaemic index), and plasma biomarkers (high-density lipoprotein cholesterol, and fasting plasma glucose). Model assumptions were assessed visually using plots of residuals. The outcome, triglyceride concentrations, was log-transformed to approximate normality. To minimise overfitting, a backward stepwise procedure was performed according to Akaike’s Information Criterion [40]. Regression model coefficients were used for dietary modelling on population-averaged food and beverage intake (grams), based on different theoretical scenarios of varying intake, and their effect on triglyceride concentration. Model goodness of fit (R^2^) and predictive accuracy (RMSE) was also reported. R version 4.1.2 was used for analysis.

## 3. Results

The mean (SD) age and BMI of the 606 Australian women of reproductive age included in the modelling dataset were 36.4 (8.2) years and 26.6 (6.3) kg/m^2^, respectively, while the median (IQR) of triglyceride was 0.9 (0.7–1.2) mmol/L (Table 2). Model coefficients used to estimate triglyceride are shown in Table S2. The final model had a root-mean-squared error of 1.1 mmol/L. The sample population mean daily intake of processed and minimally/unprocessed foods used in the modelled dietary scenarios are shown in Table 3. The average daily intakes of fruits and vegetables were equivalent to <1 serve and ~2 serves, respectively. Energy intake from all processed foods was 71% and from minimally/unprocessed foods was 24%.

### Scenario Modelling on Estimated Triglyceride Concentrations and Nutrient Profile

**Figure 1:**
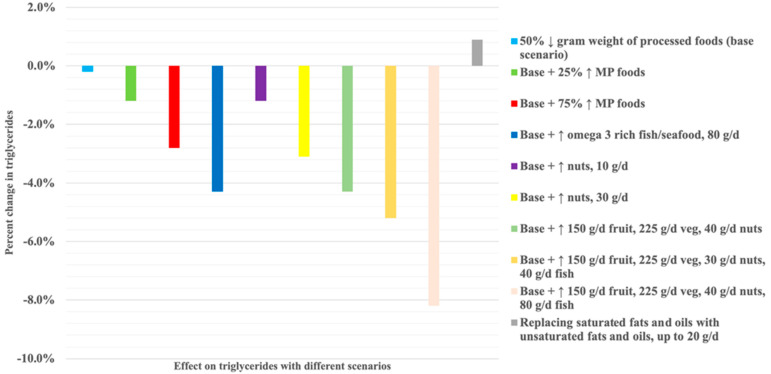
Modelled percent changes in triglycerides, compared to baseline, following a range of hypothetical scenarios. ↓ = decrease; ↑ = increase.

**Figure 2:**
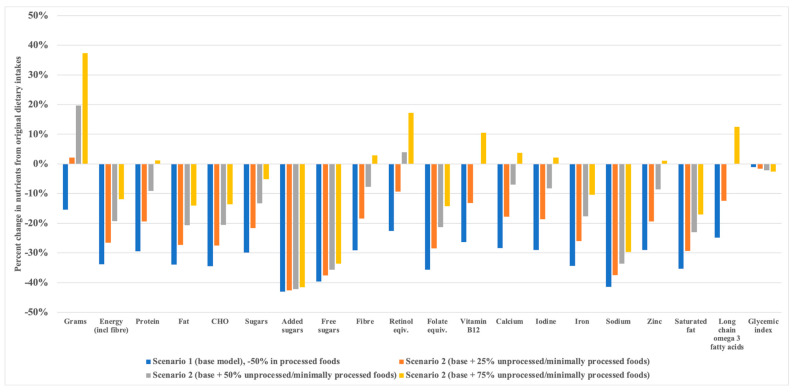
Modelled percent differences in nutrients from all food choices compared to original intake.

Predicted triglycerides and their 95% CIs following each scenario are reported in Table S3. In Scenario 1, reducing PFs by 50% resulted in a theoretical decrease in triglycerides of 0.2% (Figure 1), whereas replacing 50% of PFs with 25%, 50%, or 75% minimally/unprocessed foods (i.e., a reduction in PFs of 50% gram weight and replacing with gram equivalent of 25–75% minimally/unprocessed foods; Scenario 2, models 1, 2, and 3) led to greater estimated reductions, of −1.2% (−0.011 mmol/L; from baseline mean 0.935; 95% CI 0.893, 0.979 to 0.924; 0.873, 0.978), −2.1% (−0.020 mmol/L; from baseline mean 0.935; 95% CI 0.893, 0.979 to 0.915; 0.865, 0.968), and −2.8% (−0.026 mmol/L; from baseline mean 0.935; 95% CI 0.893, 0.979 to 0.909; 0.858, 0.963), respectively (Figure 1). Compared to baseline intakes, decreasing intake of PFs by 50% led to modelled reductions in energy by ~2600 kJ/d, and all nutrients, particularly added sugars (down to 26.5 g/d), free sugars (down to 32.0 g/d), alcohol (down to 4.5 g/d), and sodium (down to 1294.7 mg/d) (Scenario 1) (Figure 2). Reducing the same amount of PFs by 50% and replacing with 25%, 50%, or 75% of a range of minimally/unprocessed foods decreased energy intake from 850 kJ/d to 2000 kJ/d but attenuated the reduction in most nutrients. However, with a 75% increase in minimally/unprocessed foods there was a >12% increase in vitamin A (retinol equivalents), vitamin B_12_, and long-chain omega 3 fatty acids, compared to original intakes (Scenario 2, model 3). Increasing intake of minimally/unprocessed foods by 25%, 50%, or 75% had comparable reductions in added sugar, free sugars, and alcohol, with little impact on glycaemic index (Figure 2).

Scenario 3a showed that a 50% reduction in PFs along with an incremental 40 g/d increase in high-omega-3 fish/seafood (up to 200 g/d; model 1–5) reduced triglycerides by up to an estimated −9.9% (−0.093 mmol/L; from baseline mean 0.935; 95% CI 0.893, 0.979 to 0.914; 0.864, 0.967). An increase in 80 g/d high-omega-3 fish led to an estimated reduction in triglycerides of −4.3% (Figure 1), with intakes of long-chain omega 3 increasing by 1800 mg/d, total fat reducing by ~8.4 g/d, and sodium intake reducing by ~860 mg/d (Table S4). An incremental increase in nuts (Scenario 3b, model 1–4) reduced triglycerides by up to an estimated −3.9% (−0.036 mmol/L; from baseline mean 0.935; 95% CI 0.893, 0.979 to 0.915; 0.862, 0.971). Figure 1 displays the reductions in triglycerides and Table S5 reports the nutrient profile with an increase of 10 g/d and 30 g/d in nuts. Although fibre intake was estimated to be ~2–5 g lower than baseline fibre intakes, total sugar, saturated fat, and sodium were lower by more than 30%.

For Scenario 4, incremental increases in vegetables (1–5 serves) or fruits (0.5–2 serves) led to an estimated reduction in triglycerides of 0–0.5% and −0.9–2.1% (Table S3), respectively. While micronutrient intakes increased accordingly with an increase in fruits or vegetables, the overall estimated total energy intake was 5500–6000 kJ/d. Tables S6 and S7 shows the modelled nutrients following the base scenario and increasing fruit or vegetable consumption by 225 g/day.

Scenario 5 shows that decreasing PFs by 50% and increasing intake of nuts (10–40 g/d), vegetables (75–225 g/d), and fruits (75–225 g/d) led to theoretical reductions in triglycerides of −1.2% (−0.011 mmol/L) to −4.3% (−0.040 mmol/L), with the largest estimated reductions from consuming 40 g/d nuts, 150 g/d fruits, and 225 g/d vegetables (Figure 1; Table S3). Table S8 shows the related nutrient profile where estimated total energy intake is 6930 kJ/d, with an ~6 g/d increase in fibre, 12–15 g reductions in added sugars and free sugars, ~830 mg reduction in sodium, and ~6 g reduction in saturated fat intake. With the addition of 40 g/d or 80 g/d high-omega-3 fish, triglycerides were further reduced by −5.2% (−0.049 mmol/L) or −8.2% (−0.077 mmol/L) (Figure 1).

Compared to baseline intake, increasing intake of fruits by 150 g/d, vegetables by 225 g/d, nuts by 30 g/d, and high-omega-3 fish by 40 g/d with a 50% reduction in PFs resulted in an ~5 g/d increase in fibre, ~880 mg/d increase in long-chain omega 3 fatty acids, reductions in added sugars by 15 g, saturated fat by 5 g, and sodium by more than 800 mg, and a slight reduction in glycaemic index (Table 4).

In Scenario 6, substituting oils with a high SFA content with healthier oils containing high-omega-3 fatty acids, along with an incremental increase of 5–20 g/d in the intake of healthier oils, led to an elevation of up to 0.9% in triglycerides but did not result in a substantial alteration in the nutrient profile (Table S9).

## 4. Discussion

The modelling scenarios were developed to quantify feasible changes in dietary intake that could lower triglyceride concentrations in women of reproductive age. Replacing 50% of processed foods with 25–75% unprocessed/minimally processed foods minimally reduced triglycerides (up to −2.8%, −0.026 mmol/L). Replacing processed foods with different combinations of fruits (75 g/d to 300 g/d), vegetables (75 g/d to 300 g/d), nuts (10–40 g/d), and fish (40–80 g/d), could reduce triglycerides by up to an estimated −8.2% (−0.077 mmol/L). Increasing intake of high-omega-3 fish (40 g/d to 200 g/d), without increasing other foods, could induce the greatest decrease in triglycerides by up to −9.9% (−0.093 mmol/L). Replacing unhealthier oils for healthier oils, or increasing intakes of vegetables alone, appeared to have negligible effects on reducing triglycerides.

### 4.1. Strengths and Limitations

This is the first study to explore the potential impact of reducing processed foods on triglycerides in reproductive age women. The current study followed key criteria relevant to dietary modelling in terms of model structure, model performance, and transparency, included a priori covariates known to be associated with triglycerides, explored several food choices, and the complete nutrient profile. Availability of blood sample data enables modelling of triglycerides and its relationship with nutrient intake, which allows for estimation of triglyceride levels for hypothetical scenarios of population-averaged nutrient intake.

Modelling studies have inherent methodological limitations. Assumptions are to be made for statistical inferences and generalisation of findings. In this study, some assumptions were made, including that risk factor effects were assumed to be independent, which could be an overestimation, and that the model assumes a linear dose–response relationship between foods and triglycerides that may not necessarily be true. The current model does not allow for changes in population dietary intakes to be modelled simultaneously but has potential for further development. The triglyceride estimation is also limited by the number of available blood sample data within the survey subset of reproductive age women, and it was assumed that the relationship between nutrient intake and triglyceride estimated from the subset is generalisable. The baseline triglyceride level in the included population was low, and thus the changes to triglycerides we identified may be smaller than what would be observed in populations who already have cardiometabolic diseases. We performed a stepwise procedure to reduce the number of coefficients, and thus other potentially important variables, such as physical activity, were not included in the model. Further assessment such as bootstrapping may be performed in the future to assess the validity of the triglyceride model.

### 4.2. Comparison to Other Studies

A substitution modelling study examined the impact of altering dietary intake to improve lipids, in which the effect estimates in that study were in the opposite direction to what we observed. Mente et al. modelled an isocaloric replacement of 3% of energy from saturated fatty acids with different nutrients on risk markers for cardiovascular disease [41]. Replacement with an equal percentage from carbohydrates led to an estimated mean increase in triglycerides of +0.022 mmol/L, which was intermediate to the magnitude of the estimated increase in triglycerides following replacement with monounsaturated fatty acids (+0.032 mmol/L) or polyunsaturated fatty acids (+0.016 mmol/L) [41]. The modelled hazard ratio estimates of cardiovascular disease events were neutral or modestly protective. That is, the modelled hazard ratio for major CVD events when changing carbohydrate intake from the average level in the reference quintile 3 (61%E; median triglyceride 1.50 mmol/L) vs. quintile 1 (45.1%E; median triglyceride 1.52 mmol/L) was 0.995 (95% CI: 0.994 to 0.997), where the adjusted corresponding change in triglycerides was 0.0042 (95% CI: 0.0034 to 0.0048) mmol/l, per 1% of energy increment in carbohydrate intake.

In terms of ultra-processed foods, little is known about the association with lipid profile in adults. In 5636 men and women aged approximately 65 years from the PREDIMED-Plus trial, there was no significant association between triglycerides and ultra-processed foods consumed at baseline [42]. However, at the first 12 months of the intervention, compared with the lower quartile, the highest quartile of ultra-processed food consumption was positively associated with plasma triglycerides (mg/dL, β = 6.79; 95%CI = 3.66, 9.91) [43], which is an approximate 0.08 mmol/L or 4.5% change in triglyceride from baseline (and a decrease in ultra-processed foods from 160 g/d to 100 g/d at 12 months). We speculate our most feasible scenario was that which included daily incorporation of 150 g fruits, 225 g vegetables, 30 g nuts, and 40 g fish. This scenario theoretically reduced triglyceride by ~5% (−0.049 mmol/L; from baseline 0.935 mmol/L to 0.886 mmol/L). Although the mean baseline triglyceride level in our study was already low, the reduction in triglyceride level is greater than the Mente et al. substitution study [41], but similar to the lower level of triglycerides from the decrease in processed foods shown in the PREDIMED-Plus trial. Yet, the mean baseline intake of ultra-processed foods in our reproductive age cohort was much higher (874 g). Whether our modelling studies contribute to a more pronounced improvement in cardiometabolic disease risk requires investigation in clinical trials. Our modelling adds to the study by Mente et al. by utilising whole foods, rather than isocaloric replacement in nutrients, and we report potentially beneficial changes to triglycerides, which were also observed from the PREDIMED-Plus trial. The improvements in nutritional profile and the reductions in triglycerides we demonstrate are at the population level, having the potential to address several health issues in reproductive aged women.

Our modelling indicates that replacing processed foods with high-omega-3 fish could be an effective way to lower triglycerides. Including 40 g/d would lower triglycerides by an estimated 2.2% (−0.021 mmol/L) and increasing fish intake by 200 g/d would lower triglycerides by 9.9% (−0.093 mmol/L). Yet, these changes are much smaller than a meta-analysis of intervention studies whereby consuming oily fish, ranging from as little as 20 g/d up to 150 g/d, was associated with a 0.11 mmol/L reduction in plasma triglycerides [36]. The baseline intake of long-chain omega 3 fatty acids in the current study was modest, at 233 mg/d, with 51 mg of long-chain omega 3 coming from the unprocessed/minimally processed foods and 116 mg/d from processed foods, likely from fried fish. These intakes are lower than the recommended Australian Dietary Guidelines of 400 mg/d for women, and far lower than the recommended 140–280 g of fish per week [44] preferably from non-fried fish. Our results indicate that an additional 200 g high-omega-3 fish intake per day is likely to be an unachievable option for most women; however, there is still some effect on reducing triglycerides at lower fish intakes.

The scenario modelling highlights some benefits to triglycerides with increasing intake of nuts, at an intake of 30–40 g/d, or just over one serving. However, given the baseline population nut intake was only 3 g/d, incorporation of 40 g/d nuts to the average daily diet may be an unlikely feasible strategy for many women of reproductive age. Different types of nuts have different effects on triglycerides [45]. Nuts contain beneficial nutrients including monounsaturated fatty acids, fibre, minerals, vitamins, and many bioactive compounds, contributing to effects on improving endothelial function, lowering oxidative stress, and reducing lipoprotein(a) level [46]. Our modelling suggests that increasing intake of nuts, along with a reduction in processed foods, may be a suitable strategy to reduce triglycerides. However, further benefits would be obtained with incorporation of fruits, vegetables, and fish.

Replacing saturated fat oils and spreads with unsaturated oils and spreads produced minimal effects on estimated triglycerides. The rationale for this scenario was based on the lipid-lowering response when replacing saturated fats and oils with unsaturated fats [47], yet we report a slight increase in triglycerides. We acknowledge there have been changes in quantity and types of oils consumed since the time of the survey [48], and perhaps the triglyceride-lowering effect from oils may occur with higher intakes of omega 3 oils [49].

### 4.3. Possible Explanations, Implications, and Future Research

The most ideal and potentially feasible scenarios may be reducing processed foods by half, and increasing fruits (150–225 g/d), vegetables (150–225 g/d), nuts (20–30 g/d), and fish (40–80 g/d) as this led to a theoretical decrease in plasma triglycerides by up to −8.2% (−0.077 mmol/L). While increasing fruits and vegetables on their own did not demonstrate meaningful reductions to triglycerides, they contribute important vitamins, and minerals, but also energy and bulk to the diet. Practically, per day, this would be an additional 1–1.5 servings of fruits, 2–3 servings of vegetables, 1 serving of nuts, and 0.5 to 0.75 servings of fish to what the population already consumes. Reducing only processed foods by half and increasing a range of unprocessed/minimally processed foods by up to 75% was also effective in reducing triglycerides. However, there was an estimated decrease in energy of up to 2000 kJ/d. Educating to increase specific foods and food groups may help individuals make informed choices about their diet, ensuring they consume a wide variety of nutrients necessary for overall health.

Currently, there are no food-based interventions that consistently lower triglycerides. In the general adult population, intervention studies on physical activity, omega 3 foods (fish and flax), and weight loss have reduced triglycerides by up to 20%, and lipid-lowering drugs by up to 15%, while fibrates, omega 3 fatty acids, and niacin supplements have more profound effects (25–45%) [50]. The novelty of dietary modelling provides estimates of how manipulation of food intake might lower triglycerides before they are tested in an intervention. The magnitude of reduction in plasma triglycerides we estimate are small, but larger than the Mente et al. [41] study that investigated risk for cardiovascular events. There is potential for testing our modelling in a dietary intervention study in sub-groups of women of reproductive age, for example, those with infertility, polycystic ovary syndrome, or gestational diabetes, as a potential strategy to mitigate risk for cardiometabolic diseases.

## 5. Conclusions

The present study highlights that increasing consumption of high-omega-3 fish may be beneficial in reducing triglycerides. However, a holistic approach of increasing intakes of fruits, vegetables, nuts, and high-omega-3 fish may be the most feasible. There was an increased effect on estimated triglycerides when replacing saturated fat oils and spreads with unsaturated options. Future dietary modelling studies could test the implications for lowering triglycerides on cardiometabolic diseases in women of reproductive age.

## Figures and Tables

**Table 1:** Rationale for the dietary scenarios and models.

Scenario	Strategy	Rationale
1 (base model)	Reducing NOVA processed and ultra-processed foods (PFs): the base model.	Based on current intake of discretionary choices in Australian diets being twice as high than recommendations [33] and results from our previous simulation modelling among Australian women of reproductive age that showed 56.2% of energy intake was consumed from processed foods contributing 60.1% SFAs, 82.7% added sugar, and 82.7% sodium [27]. A 50% reduction in PFs may be a simple approach to reduce energy intake, processed foods, and discretionary choices, thereby reducing the detrimental impact on cardiometabolic health. This is the base model and is included in all subsequent scenarios except Scenario 6 which is the replacement of oils.
2	Scenario 1 plus replacing PFs with NOVA unprocessed/minimally processed foods.	The Australian diet is typically low in unprocessed/minimally processed foods such as vegetables, fruits, cereals, milk/fermented milks, poultry, fish, and seafood that are important sources of healthy nutrients [34].
3	Scenario 1 plus replacing PFs with high-omega-3 foods.	Although nuts and fatty fish that are rich in omega 3 fatty acids have been shown to reduce triglycerides [35,36], intake in the Australian population is suboptimal [33].
4	Scenario 1 plus replacing PFs with fruits and vegetables.	Higher intakes of dietary fibre have been associated with lower triglyceride levels [37]. Vegetables and fruits are two major sources of fibre but less than a third (28.2%) of Australian adults consume sufficient amounts of fibre [33], and a majority of women of reproductive age do not meet the recommended intake for vegetables (85.4%) or fruits (73.8%) [38].
5	Scenario 1 plus replacing PFs with different combinations of food choices in Scenarios 2–4.	Examining different combinations of potentially feasible increases in healthier foods such as nuts, vegetables, and fruits.
6	Replacing higher saturated fatty acid oils with higher poly-and/or monounsaturated fatty acid oils.	Compared to SFAs, intake of monounsaturated fats (e.g., α-linolenic acid and oleic acid) and polyunsaturated fats found in flaxseed, olive, canola, and sesame oils has been shown to reduce triglyceride levels [39].

**Table 2:** Demographics of the study participants (*n* = 606).

Study Variable	Included Participants
Age (years): Mean (SD)	36.38 (8.16)
BMI (kg/m^2^): Mean (SD)	26.56 (6.32)
HDL-C (mmol/L): Mean (SD)	1.47 (0.36)
Fasting plasma glucose (mmol/L): Mean (SD)	4.85 (0.71)
Triglycerides (mmol/L): Median (IQR)	0.90 (0.70–1.20)
Smoking status: n (%)	
Never smoked	362 (59.74)
Ex-smoker	165 (27.23)
Current smoker	79 (13.04)
Country of birth: n (%)	
Australia/New Zealand	443 (73.1)
Other	163 (26.9)
Family history of diabetes: n (%)	
No	427 (70.46)
Yes	179 (29.54)

SD: Standard deviation; IQR: interquartile range.

**Table 3:** Population intakes of NOVA food groups and the food groups used in the different scenarios.

Nutrient	All Food Intake, Baseline	Total PFs ^1^	MP ^2^	PCI ^3^	Fruits	Vegetables	Nuts	High-Omega-3 Fish ^4^
Gram weight (g)	3113	874	1898	13.6	211	111	3.0	2.2
Energy including fibre (kJ)	7661	5110	245	1470	451	290	71.6	24.5
Protein (g)	79.1	46.6	0.1	26.6	1.4	3.3	0.5	0.6
Fat (g)	67.1	45.5	3.6	13	0.5	2.5	1.5	0.4
Carbohydrates (g)	202	136	6.8	30.6	22.3	6.6	0.2	0.0
Sugars (g)	94	52.3	6.8	11.5	20.9	2.5	0.1	0.0
Added sugars (g)	46.5	37.2	5.6	0.6	3.0	0.0	0.0	0.0
Free sugars (g)	53	38.5	6.8	0.7	7.0	0.0	0.0	0.0
Fibre (g)	20.6	11.7	0.0	2.1	3.4	3.0	0.3	0.0
Alcohol (g)	9.1	9.1	0.0	0.0	0.0	0.0	0.0	0.0
Retinol equivalents (µg)	779	344	14.1	120	71.2	229	0.1	1.3
Total folate equivalents (µg)	532	377	72.3	0.2	42.2	38.6	2.0	0.0
Vitamin B_12_ (µg)	3.8	1.9	1.8	0.0	0.0	0.1	0.0	0.0
Calcium (mg)	761	430	268	2.3	22.5	32.9	5.1	0.3
Iodine (µg)	153	87.3	59.4	0.4	3.1	2.2	0.0	0.5
Iron (mg)	9.6	6.5	1.7	0.0	0.5	0.7	0.1	0.0
Sodium (mg)	2210	1827	290	32.4	10.1	49.5	0.3	1.5
Zinc (mg)	9.3	5.5	2.9	0.0	0.2	0.5	0.1	0.0
Saturated fat (g)	25.2	17.9	5.3	1.1	0.0	0.6	0.2	0.1
Monounsaturated fat (g)	25.5	17	4.9	1.4	0.1	1.2	0.7	0.2
Linoleic acid (g)	8.8	5.9	1.2	0.7	0.1	0.4	0.5	0.0
Alpha linolenic acid (g)	1.3	0.9	0.1	0.1	0.0	0.0	0.1	0.0
Long-chain omega 3 fatty acids (mg)	233.1	115.7	56.9	1.5	0.0	7.6	0.0	51.4
Glycaemic Index	54.8	56.1	52.3	63.2	47.1	57.6	0.0	0.0
Glycaemic Load	110	76	16	4.3	10.5	3.8	0.0	0.0

^1^ Including ultra-processed and processed foods; ^2^ Unprocessed/minimally processed foods; ^3^ Processed culinary ingredients; ^4^ Including poultry, fish, and seafood.

**Table 4:** Modelled nutrients following a reduction in PFs by 50% and increasing fruits by 150 g/d, vegetables by 225 g/d, nuts by 30 g/d, and high-omega-3 fish by 40 g/d.

Nutrient	Modelled Intakes	Total PFs ^1^	MP ^2^	PCI ^3^	Fruits	Vegetables	Nuts	High-Omega-3 Fish ^4^
Energy including fibre (kJ)	7140	2555	1470	245	931	681	788	470
Protein (g)	77.7	23.3	26.6	0.1	2.9	7.8	5.9	11.1
Fat (g)	70.7	22.7	13	3.6	1.0	6.0	16.7	7.6
CHO (g)	169	67.8	30.6	6.8	46	15.6	2.3	0.0
Sugars (g)	94.4	26.2	11.5	6.8	43.1	5.8	1.1	0.0
Added sugars (g)	31.1	18.6	0.6	5.6	6.3	0.0	0.0	0.0
Free sugars (g)	41.4	19.3	0.7	6.8	14.6	0.0	0.1	0.0
Fibre (g)	25.7	5.9	2.1	0.0	7.0	7.0	3.7	0.0
Alcohol (g)	4.5	4.5	0.0	0.0	0.0	0.0	0.0	0.0
Retinol equivalents (µg)	1017	172	120	14.1	147	537	0.9	25.9
Total folate equivalents (µg)	461	188	72.3	0.2	87.2	90.7	21.7	0.7
Vitamin B_12_ (µg)	3.8	0.9	1.8	0.0	0.0	0.1	0.0	0.9
Calcium (mg)	670	215	268	2.3	46.4	77.2	55.9	5.1
Iodine (µg)	125	43.7	59.4	0.4	6.3	5.2	0.4	9.6
Iron (mg)	9.5	3.2	1.7	0.0	1.0	1.7	1.3	0.5
Sodium (mg)	1404	913	290	32.4	20.8	116	3.6	28
Zinc (mg)	9.0	2.7	2.9	0.0	0.5	1.3	1.3	0.3
Saturated fat (g)	20.4	8.9	5.3	1.1	0.1	1.5	1.7	1.7
Monounsaturated fat (g)	28.9	8.5	4.9	1.4	0.2	2.8	7.9	3.1
Linoleic acid (g)	12.2	3.0	1.2	0.7	0.2	0.8	5.7	0.6
Alpha linolenic acid (g)	1.7	0.5	0.1	0.1	0.0	0.1	0.7	0.2
Long-chain omega 3 fatty acids (mg)	1121	57.9	56.9	1.5	0.0	17.9	0.0	987
Glycaemic Index	52.9	56.0	52.3	63.2	47.2	57.7	21.7	0.0
Glycaemic Load	89.5	38	16	4.3	21.7	9	0.5	0.0

^1^ Including ultra-processed and processed foods; ^2^ Unprocessed/minimally processed foods; ^3^ Processed culinary ingredients; ^4^ ≥800 mg long chain omega 3 fatty acids per 100 g.

## Data Availability

Data availability and access can be found from the following URL: https://www.abs.gov.au/statistics/microdata-tablebuilder/datalab (accessed on 16 June 2021). Sources of detailed microdata in the DataLab were obtained from: the Australian Bureau of Statistics (2011–2013) NHS14E National Health Survey 2014–2015 Expanded; AHS11E Australian Health Survey, Core Content—Risk Factors and Selected Health Conditions, 2011–2012; and the NPAS11E Australian Health Survey, Nutrition and Physical Activity, 2011–2012 (ABS DataLab), accessed during 2022 and 2023. Access to microdata requires a project proposal submitted to the Australian Bureau of Statistics for consideration and approval.

## Supplementary Materials

The following supporting information can be downloaded at: https://www.mdpi.com/article/10.3390/nu15245137/s1, Figure S1: Participant flow; Table S1: Characteristics of included and excluded participants; Table S2: Model coefficients from the triglycerides model; Table S3: Predicted triglycerides and their 95% CI’s following each scenario; Table S4: Estimated nutrient profile following a decrease in PF1 by 50% and increasing high omega 3 fish by 80 g/d; Table S5: Estimated nutrient profile following a decrease in PF1 by 50% and increasing nuts by 10 g/d and 30 g/d; Table S6: Modelled nutrients following a reduction in PF1 by 50% and increasing fruit consumption by 225 g/day; Table S7: Modelled nutrients following a reduction in PF1 by 50% and increasing vegetable consumption by 225 g/day; Table S8: Modelled nutrients following a reduction in PF by 50% and increasing fruit by 150 g/d, vegetables by 225 g/d, and nuts by 40 g/d; Table S9: Modelled nutrients when replacing oils containing high level of SFA with oils containing high omega 3 FAs.
