# Supplementary material for: Dietary Strategies to Reduce Triglycerides in Women of Reproductive Age: A Simulation Modelling Study
Source: Nutrients. 2023 Dec 18;15(24):5137. doi: 10.3390/nu15245137 (PMC10745529; doi:10.3390/nu15245137)
Supplement: Supplementary file 1 [file nutrients-15-05137-s001.zip › nutrients-2747243-supplementary.pdf]

Supplementary files (intended for publication)

Figure S1: Participant flow.

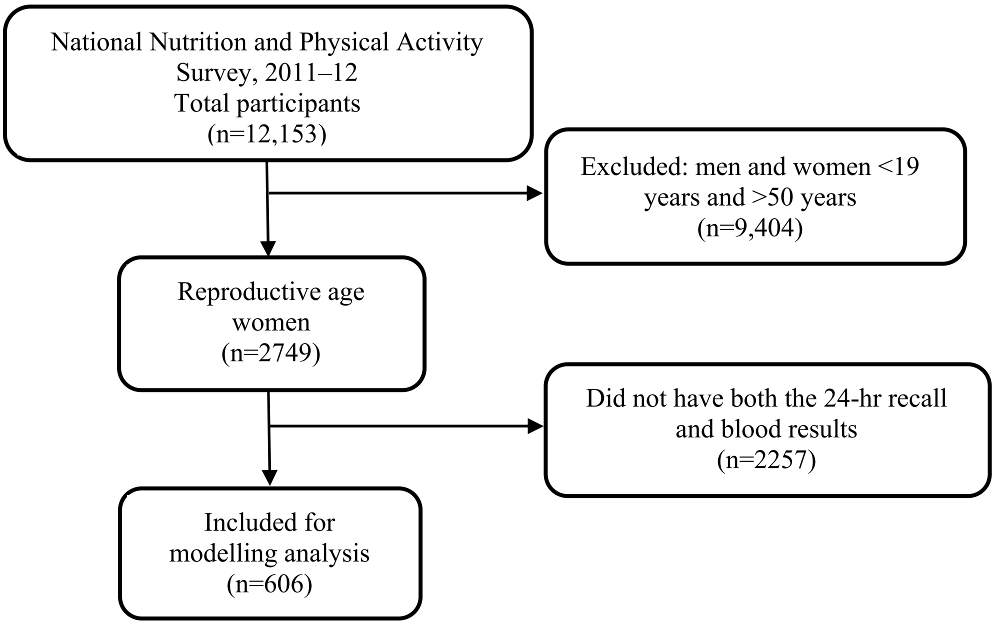

Table S1: Characteristics of included and excluded participants.

	**Included participants (n=606)**	**Excluded participants (n=1540)**
Age (years): Mean (SD)	36.38 (8.16)	34.86 (8.62)
BMI (kg/m^2): Mean (SD)	26.56 (6.32)	26.63 (6.11)
HDL (mmol/L): Mean (SD)	1.47 (0.36)	-
Fasting plasma glucose (mmol/L): Mean (SD)	4.85 (0.71)	-
Triglycerides (mmol/L): Median (IQR)	0.90 (0.70 – 1.20)	1.00 (0.80 – 1.30)
Smoking status: n (%)		
Never smoked	362 (59.74)	827 (53.7)
Ex-smoker	165 (27.23)	335 (21.75)
Current smoker	79 (13.04)	378 (24.55)
Country of birth: n (%)		
Australia/New Zealand	443 (73.1)	1195 (77.6)
Other	163 (26.9)	345 (22.4)
Family history of diabetes: n (%)		
No	427 (70.46)	1136 (73.77)
Yes	179 (29.54)	404 (26.23)

SD: standard deviation; IQR: interquartile range

Table S2: Model coefficients from the triglycerides model.

Variable	Coefficients
(Intercept)	-1.0190
Glycemic index	0.0049
Smoking status: ex-smoker	0.0374
Smoking status: current smoker	0.2110
Body mass index	0.0160
High density lipoprotein cholesterol	-0.1945
Glucose	0.1184
Energy	-0.0002
Carbohydrate without sugar and alcohol	0.0126
Starch	-0.0093
Sugar	-0.0108
Alcohol	0.0044
Retinol equivalents	0.0000
Vitamin B1	-0.0050
Vitamin B2	0.0059
Vitamin B3	0.0021
Folic acid	-0.0002
Vitamin B6	0.0013
Iodine	0.0004
Potassium	0.0001
Caffeine	-0.0003
Saturated fat	0.0131
Monounsaturated fat	0.0065
Trans fatty acids	-0.0001

Table S3: Predicted triglycerides and their 95% CI's following each scenario

Scenario	Predicted triglyceride level (95% CI)
Baseline, original intakes	0.935 (0.893, 0.979)
Scenario 1. Reduce NOVA processed and ultra-processed foods (PF): the base model	0.933 (0.878, 0.991)
Scenario 2, model 1. Base model + replace PF with NOVA unprocessed/minimally processed foods, by 25%	0.924 (0.873, 0.978)
Scenario 2, model 2. Base model + replace PF with NOVA unprocessed/minimally processed foods, by 50%	0.915 (0.865, 0.968)
Scenario 2, model 3. Base model + replace PF with NOVA unprocessed/minimally processed foods, by 75%	0.909 (0.858, 0.963)
Scenario 3a, model 1. Base model + replace PF with high omega 3 fish, by 40 g/d	0.914 (0.864, 0.967)
Scenario 3a, model 2. Base model + replace PF with high omega 3 fish, by 80 g/d	0.895 (0.846, 0.947)
Scenario 3a, model 3. Base model + replace PF with high omega 3 fish, by 120 g/d	0.878 (0.827, 0.931)
Scenario 3a, model 4. Base model + replace PF with high omega 3 fish, by 160 g/d	0.860 (0.806, 0.918)
Scenario 3a, model 5. Base model + replace PF with high omega 3 fish, by 200 g/d	0.842 (0.783, 0.906)
Scenario 3b, model 1. Base model + replace PF with nuts, by 10g/d	0.924 (0.871, 0.980)
Scenario 3b, model 2. Base model + replace PF with nuts, by 20g/d	0.915 (0.862, 0.971)
Scenario 3b, model 3. Base model + replace PF with nuts, by 30g/d	0.906 (0.853, 0.963)
Scenario 3b, model 4. Base model + replace PF with nuts, by 40g/d	0.899 (0.844, 0.958)
Scenario 4a, model 1. Base model + increase gram intake of vegetables by 75 g/d	0.935 (0.880, 0.993)
Scenario 4a, model 2. Base model + increase gram intake of vegetables by 150 g/d	0.936 (0.880, 0.995)
Scenario 4a, model 3. Base model + increase gram intake of vegetables by 225 g/d	0.936 (0.879, 0.997)
Scenario 4a, model 4. Base model + increase gram intake of vegetables by 300 g/d	0.940 (0.881, 1.003)
Scenario 4b, model 1. Base model + increase gram intake of fruit by 75 g/d	0.927 (0.873, 0.984)
Scenario 4b, model 2. Base model + increase gram intake of fruit by 150 g/d	0.922 (0.868, 0.979)
Scenario 4b, model 3. Base model + increase gram intake of fruit by 225 g/d	0.920 (0.865, 0.978)
Scenario 4b, model 4. Base model + increase gram intake of fruit by 300 g/d	0.915 (0.859, 0.975)
Scenario 5, model 1. Base scenario + increase fruit by 225 g/d, vegetables by 225 g/d, nuts by 10 g/d	0.924 (0.864, 0.988)
Scenario 5, model 2. Base scenario + increase fruit by 225 g/d, vegetables by 225 g/d, nuts by 20 g/d	0.916 (0.856, 0.980)
Scenario 5, model 3. Base scenario + increase fruit by 150 g/d, vegetables by 150 g/d, nuts by 10 g/d	0.922 (0.866, 0.981)
Scenario 5, model 4. Base scenario + increase fruit by 150 g/d, vegetables by 225 g/d, nuts by 20 g/d	0.912 (0.855, 0.972)
Scenario 5, model 5. Base scenario + increase fruit by 150 g/d, vegetables by 225 g/d, nuts by 30 g/d	0.903 (0.847, 0.964)
Scenario 5, model 6. Base scenario + increase fruit by 150 g/d, vegetables by 225 g/d, nuts by 40 g/d	0.895 (0.836, 0.957)
Scenario 5, model 7. Base scenario + increase fruit by 150 g/d, vegetables by 150 g/d, nuts by 40 g/d	0.897 (0.840, 0.959)
Scenario 5, model 8. Base scenario + increase fruit by 150 g/d, vegetables by 150 g/d, nuts by 30 g/d	0.906 (0.850, 0.966)
Scenario 6. Replace oils with high SFA content (e.g. butter, dairy blend) with healthier alternatives (e.g. flaxseed, olive, canola, and sesame oils)	0.933 (0.878, 0.991)
Scenario 7, model 1. Base scenario + increase fruit by 150 g/d, vegetables by 225 g/d, nuts by 30 g/d, high omega 3 fish by 40 g/d	0.886 (0.830, 0.945)
Scenario 7, model 2. Base scenario + increase fruit by 150 g/d, vegetables by 225 g/d, nuts by 30 g/d, high omega 3 fish by 80 g/d	0.868 (0.812, 0.927)
Scenario 7, model 3. Base scenario + increase fruit by 150 g/d, vegetables by 225 g/d, nuts by 30 g/d, high omega 3 fish by 120 g/d	0.850 (0.791, 0.913)

Scenario 7, model 4. Base scenario + increase fruit by 150 g/d, vegetables by 225 g/d, nuts by 40 g/d, high omega 3 fish by 40 g/d	0.877 (0.820, 0.939)
Scenario 7, model 5. Base scenario + increase fruit by 150 g/d, vegetables by 225 g/d, nuts by 40 g/d, high omega 3 fish by 80 g/d	0.858 (0.799, 0.921)
Scenario 7, model 6. Base scenario + increase fruit by 150 g/d, vegetables by 225 g/d, nuts by 40 g/d, high omega 3 fish by 120 g/d	0.841 (0.779, 0.908)

Table S4: Estimated nutrient profile following a decrease in PF[1] by 50% and increasing high omega 3 fish by 80 g/d.

Nutrient	All food intake, baseline	Modelled intakes	Total PF[1]	MP[2]	PCI[3]	Fish (>800 mg/100 g)
Energy including fibre (kJ)	7661.4	5961.3	2590.6	2210.7	245.0	915.0
Protein (g)	79.1	76.8	23.4	31.8	0.1	21.7
Fat (g)	67.1	58.7	22.8	17.5	3.6	14.8
CHO (g)	203.0	133.1	69.9	56.3	6.8	0.0
Sugars (g)	94.0	65.9	28.1	31.1	6.8	0.0
Added sugars (g)	46.5	26.5	20.0	0.9	5.6	0.0
Free sugars (g)	53.0	32.0	21.0	4.2	6.8	0.0
Fibre (g)	20.6	14.6	5.9	8.7	0.0	0.0
Alcohol (g)	9.1	4.5	4.5	0.0	0.0	0.0
Retinol equivalents (µg)	779.1	652.4	175.7	412.1	14.1	50.4
Total folate equivalents (µg)	532.0	343.6	189.8	152.2	0.2	1.4
Vitamin B12 (µg)	3.8	4.6	0.9	1.8	0.0	1.8
Calcium (mg)	760.8	554.1	216.3	325.6	2.3	9.9
Iodine (µg)	153.0	126.7	44.4	63.2	0.4	18.7
Iron (mg)	9.6	7.3	3.3	3.0	0.0	1.0
Sodium (mg)	2210.2	1347.7	915.5	345.3	32.4	54.5
Zinc (mg)	9.3	7.1	2.7	3.8	0.0	0.6
Saturated fat (g)	25.2	19.5	9.0	6.1	1.1	3.3
Monounsaturated fat (g)	25.5	22.9	8.5	7.0	1.4	6.1
Linoleic acid (g)	8.8	7.0	3.0	2.1	0.7	1.2
Alpha linolenic acid (g)	1.3	1.2	0.5	0.2	0.1	0.4
Long chain omega 3 fatty acids (mg)	233.1	2045.4	57.9	64.5	1.5	1921.6
Glycemic Index	54.8	54.1	55.9	50.9	63.2	0.0
Glycemic Load	110.7	71.7	39.0	28.4	4.3	0.0

[1]Including ultra-processed and processed foods; [2]Unprocessed/minimally processed foods other than high omega 3 fish; [3]Processed culinary ingredients

Table S5: Estimated nutrient profile following a decrease in PF[1] by 50% and increasing nuts by 10 g/d and 30 g/d.

Nutrient	Modelled intakes	Nuts, 10 g/d	Modelled intakes	Nuts, 30 g/d
Energy including fibre (kJ)	5309.5	310.3	5786.8	787.6
Protein (g)	57.6	2.3	61.1	5.9
Fat (g)	49.3	6.6	59.5	16.7
CHO (g)	133.8	0.9	135.2	2.3
Sugars (g)	66.3	0.4	67.0	1.1
Added sugars (g)	26.5	0.0	26.5	0.0
Free sugars (g)	32.1	0.0	32.1	0.1
Fibre (g)	15.8	1.4	18.0	3.7
Alcohol (g)	4.5	0.0	4.5	0.0
Retinol equivalents (μg)	603.6	0.4	604.2	0.9
Total folate equivalents (μg)	348.8	8.5	362.0	21.7
Vitamin B12 (μg)	2.8	0.0	2.8	0.0
Calcium (mg)	561.4	22.0	595.3	55.9
Iodine (μg)	108.7	0.1	108.9	0.4
Iron (mg)	6.7	0.5	7.5	1.3
Sodium (mg)	1295.8	1.4	1298.0	3.6
Zinc (mg)	7.0	0.5	7.8	1.3
Saturated fat (g)	16.8	0.7	17.8	1.7
Monounsaturated fat (g)	19.4	3.1	24.2	7.9
Linoleic acid (g)	7.6	2.2	11.0	5.7
Alpha linolenic acid (g)	1.0	0.3	1.5	0.7
Long chain omega 3 fatty acids (mg)	175.3	0.0	175.3	0.0
Glycemic Index	53.9	22.2	53.6	21.7
Glycemic Load	71.8	0.2	72.1	0.5

[1]Including ultra-processed and processed foods

Table S6: Modelled nutrients following a reduction in PF[1] by 50% and increasing fruit consumption by 225 g/day.

Nutrient	Modelled intakes	Total PF[1]	MP[2]	PCI[3]	Fruit
Energy including fibre (kJ)	5690	2554.9	1855.8	245.0	1034.3
Protein (g)	57.6	23.3	31.1	0.1	3.2
Fat (g)	45.0	22.7	17.5	3.6	1.2
CHO (g)	164.5	67.9	37.6	6.8	52.2
Sugars (g)	94.9	26.2	14.1	6.8	47.9
Added sugars (g)	31.8	18.6	0.7	5.6	7.0
Free sugars (g)	43	19.3	0.7	6.8	16.2
Fibre (g)	19.1	5.9	5.4	0.0	7.8
Alcohol (g)	4.5	4.5	0.0	0.0	0.0
Retinol equivalents (μg)	699.1	172.1	349.5	14.1	163.2
Total folate equivalents (μg)	398.3	188.3	113	0.2	96.9
Vitamin B12 (μg)	2.8	0.9	1.9	0.0	0.0
Calcium (mg)	575	214.9	306.3	2.3	51.5
Iodine (μg)	113.3	43.7	62.1	0.4	7.0
Iron (mg)	7.0	3.2	2.6	0.0	1.1
Sodium (mg)	1310.1	913.1	341.6	32.4	23.1
Zinc (mg)	6.9	2.7	3.6	0.0	0.5
Saturated fat (g)	16.4	8.9	6.2	1.1	0.1
Monounsaturated fat (g)	17.2	8.5	7.0	1.4	0.3
Linoleic acid (g)	6.0	3.0	2.1	0.7	0.2
Alpha linolenic acid (g)	0.8	0.5	0.2	0.1	0.0
Long chain omega 3 fatty acids (mg)	175.3	57.9	115.9	1.5	0.0
Glycemic Index	52.9	56.0	53.0	63.2	47.1
Glycemic Load	86.3	38.0	19.9	4.3	24.1

[1]Including ultra-processed and processed foods; [2]Unprocessed/minimally processed foods; [3]Processed culinary ingredients

Table S7: Modelled nutrients following a reduction in PF[1] by 50% and increasing vegetable consumption by 225 g/day.

Nutrient	Modelled intakes	Total PF[1]	MP[2]	PCI[3]	Vegetables
Energy including fibre (kJ)	5574.5	2590.6	1879.0	245.0	859.8
Protein (g)	60.9	23.4	28.7	0.1	8.7
Fat (g)	47.9	22.8	15.3	3.6	6.2
CHO (g)	147.3	69.9	46.3	6.8	24.3
Sugars (g)	74.1	28.1	25.3	6.8	13.9
Added sugars (g)	26.5	20.0	0.9	5.6	0.0
Free sugars (g)	36.7	21.0	0.9	6.8	8.0
Fibre (g)	19.4	5.9	5.3	0.0	8.1
Alcohol (g)	4.5	4.5	0.0	0.0	0.0
Retinol equivalents (μg)	954.9	175.7	164.9	14.1	600.1
Total folate equivalents (μg)	420.2	189.8	97.2	0.2	133.1
Vitamin B12 (μg)	2.9	0.9	1.8	0.0	0.1
Calcium (mg)	598.2	216.3	287.9	2.3	91.7
Iodine (μg)	112.9	44.4	60.6	0.4	7.5
Iron (mg)	7.5	3.3	2.2	0.0	1.9
Sodium (mg)	1368	915.5	294.9	32.4	125.2
Zinc (mg)	7.4	2.7	3.2	0.0	1.4
Saturated fat (g)	17.2	9.0	5.6	1.1	1.5
Monounsaturated fat (g)	18.7	8.5	5.9	1.4	2.9
Linoleic acid (g)	6.4	3.0	1.8	0.7	0.9
Alpha linolenic acid (g)	0.9	0.5	0.2	0.1	0.1
Long chain omega 3 fatty acids (mg)	186.0	57.9	108.3	1.5	18.4
Glycemic Index	54.1	55.9	50.2	63.2	54.3
Glycemic Load	79.3	39.0	23.0	4.3	13.1

[1]Including ultra-processed and processed foods; [2]Unprocessed/minimally processed foods; [3]Processed culinary ingredients

Table S8: Modelled nutrients following a reduction in PF by 50% and increasing fruit by 150 g/d, vegetables by 225 g/d, and nuts by 40 g/d.

Nutrient	Modelled intakes	Total PF[1]	MP[2]	PCI[3]	Fruit	Vegetables	Nuts
Energy including fibre (kJ)	6932.9	2554.9	1494.1	245	931.3	681.4	1026.2
Protein (g)	68.9	23.3	27.2	0.1	2.9	7.8	7.7
Fat (g)	68.6	22.7	13.4	3.6	1	6	21.8
CHO (g)	171.2	67.9	30.6	6.8	47	15.7	3
Sugars (g)	94.8	26.2	11.5	6.8	43.1	5.8	1.4
Added sugars (g)	31.1	18.6	0.6	5.6	6.3	0	0
Free sugars (g)	41.5	19.3	0.7	6.8	14.6	0	0.1
Fibre (g)	26.8	5.9	2.1	0	7	7	4.8
Alcohol (g)	4.5	4.5	0.0	0.0	0.0	0.0	0.0
Retinol equivalents (µg)	992.2	172.1	120.9	14.1	147	536.8	1.2
Total folate equivalents (µg)	467.1	188.3	72.4	0.2	87.2	90.7	28.3
Vitamin B12 (µg)	2.9	0.9	1.8	0	0	0.1	0
Calcium (mg)	682	214.9	268.4	2.3	46.4	77.2	72.9
Iodine (µg)	116	43.7	59.9	0.4	6.3	5.2	0.5
Iron (mg)	9.5	3.2	1.8	0	1	1.7	1.7
Sodium (mg)	1378.9	913.1	291.8	32.4	20.8	116.2	4.7
Zinc (mg)	9.2	2.7	2.9	0	0.5	1.3	1.7
Saturated fat (g)	19.3	8.9	5.4	1.1	0.1	1.5	2.2
Monounsaturated fat (g)	28.3	8.5	5.1	1.4	0.2	2.8	10.3
Linoleic acid (g)	13.3	3	1.2	0.7	0.2	0.8	7.4
Alpha linolenic acid (g)	1.8	0.5	0.1	0.1	0	0.1	1
Long chain omega 3 fatty acids (mg)	185.6	57.9	108.3	1.5	0	17.9	0
Glycemic Index	52.8	56.0	52.3	63.2	47.2	57.7	20.0
Glycemic Load	91.9	38.0	16.0	4.3	21.7	11.6	0.3

[1]Including ultra-processed and processed foods; [2]Unprocessed/minimally processed foods; [3]Processed culinary ingredient

Table S9: Modelled nutrients when replacing oils containing high level of SFA with oils containing high omega 3 FAs.

Nutrient	Replacement with 5 g/d of high omega 3 oils	Replacement with 10 g/d of high omega 3 oils	Replacement with 15 g/d of high omega 3 oils	Replacement with 20 g/d of high omega 3 oils
Energy including fibre (kJ)	5230.3	5393.1	5555.9	5718.7
Protein (g)	55.8	55.8	55.8	55.8
Fat (g)	48.6	53.0	57.4	61.8
CHO (g)	133.1	133.1	133.1	133.1
Sugars (g)	65.9	65.9	65.9	65.9
Added sugars (g)	26.5	26.5	26.5	26.5
Free sugars (g)	32.0	32.0	32.0	32.0
Fibre (g)	14.6	14.6	14.6	14.6
Alcohol (g)	4.5	4.5	4.5	4.5
Retinol equivalents (μg)	604.7	607.0	609.3	611.6
Total folate equivalents (μg)	342.2	342.2	342.3	342.3
Vitamin B12 (μg)	2.8	2.8	2.8	2.8
Calcium (mg)	544.5	544.5	544.5	544.5
Iodine (μg)	108.6	108.6	108.6	108.6
Iron (mg)	6.3	6.3	6.3	6.3
Sodium (mg)	1295.2	1296.4	1297.6	1298.7
Zinc (mg)	6.6	6.6	6.6	6.6
Saturated fat (g)	16.9	17.6	18.2	18.9
Monounsaturated fat (g)	20.0	22.9	25.9	28.9
Linoleic acid (g)	6.3	6.7	7.1	7.6

Alpha linolenic acid (g)	0.9	1.1	1.2	1.3
Long chain omega 3 fatty acids (mg)	175.2	175.2	175.2	175.2
Glycemic Index	54.1	54.1	54.1	54.1
Glycemic Load	71.7	71.7	71.7	71.7
